# Experimental and Computational Study on Inhibitory Effect and Adsorption Properties of *N*-Acetylcysteine Amino Acid in Acid Environment

**DOI:** 10.3390/molecules28196799

**Published:** 2023-09-25

**Authors:** Adriana Samide, Aurelian Dobriţescu, Cristian Tigae, Cezar Ionuţ Spînu, Bogdan Oprea

**Affiliations:** 1 Department of Chemistry, Faculty of Sciences, University of Craiova, 107i Calea Bucuresti, 200478 Craiova, Romania; cristian.tigae@edu.ucv.ro (C.T.); cezar.spinu@edu.ucv.ro (C.I.S.); 2 Faculty of Medicine, University of Medicine and Pharmacy, Petru Rares, 2, 200349 Craiova, Romania; bogdan.oprea@umfcv.ro

**Keywords:** *N*-acetylcysteine, corrosion inhibitor, electrochemical measurements, DFT, Monte Carlo simulation

## Abstract

Potentiodynamic polarization (PDP) and electrochemical impedance spectroscopy (EIS) were applied to study the inhibitory effect of *N*-acetylcysteine (NAC) on corrosion inhibition of carbon steel in hydrochloric acid solution. *N*-acetylcysteine influenced the iron dissolution to a greater extent than the hydrogen evolution reaction acting as a mixed inhibitor, predominantly anodic. The charge transfer resistance (R_ct_) gradually increased with the inhibitor concentration. From both methods, the inhibition efficiency (IE) reached a value of 89 ± 1% and NAC adsorption followed the Temkin isotherm. The value of adsorption Gibbs energy (Δ$G_{\mathrm{ads}}^{o}$), around −35 kJ mol^−1^, indicated a spontaneous adsorption and mixed action mechanism, with NAC chemical adsorption prevailing over physical one. New data will be reported by the computational study, that was performed using the density functional theory (DFT) method in aqueous phase. Quantum chemical descriptors were determined by B3LYP theory level with 6–31G+(d) basis set. Metropolis Monte Carlo atomistic simulation was used to reveal the adsorption configuration and interactions between acetylcysteine molecules and the carbon steel surface. Theoretical results were consistent with the experimental data, showing that the inhibitor action mechanism consisted of mainly chemisorption of its molecules on the carbon steel surface accompanied by van der Waals forces and electrostatic interactions.

## 1. Introduction

Corrosion control and prevention are intensively practiced to reduce the damage to metals/alloys and implicitly to increase their lifetime, to protect the environment and to reduce the economic impact [1,2] imposed by the replacement of industrial aggregates affected by generalized, localized or pitting corrosion. Carbon steel has a high application potential in many industrial branches due to its processing and adaptation performance generated by good mechanical properties and fair resistance in various media, as well as its relatively low costs [3,4], but it becomes susceptible to corrosive attack when in contact with aggressive environments, especially those containing chloride anions [5,6].

A varied range of anticorrosion systems were developed by modeling of protective layers at the metal/medium interface through the adsorption of environmentally friendly inhibitors such as drugs [7,8,9], plant extracts [10], prepolymers/polymers [11,12,13,14,15] and amino acids [6,16,17,18,19,20,21,22,23,24,25,26,27,28,29,30,31]. The corrosion inhibitors contain in their molecular structure heteroatoms such as oxygen, sulfur, nitrogen and phosphorus, possessing lone electron pairs that can be easily delivered towards the iron vacant d-orbital, attaching on the substrate by strong bonds.

Recent studies were focused on the investigation of cysteine as a single corrosion inhibitor for low/mild carbon steel in the presence of Cl^−^ ions [4,16,17] or in a mixture with certain surfactants [18] to improve the inhibition performance.

*N*-acetylcysteine (NAC) is a derivative of L-cysteine (acetylated cysteine) and a precursor of glutathione. It is known for its oxidizing and anti-inflammatory activity [32,33] and as an antidote for paracetamol overdose [32]. Also, NAC was evaluated for its neuroprotective potential with the aim of preventing the onset of dementia [33] or as a possible contributor in adjuvant therapy associated with SARS-CoV-2 infection [34]. Due to its environmentally friendly qualities, NAC was studied as a corrosion inhibitor of different types of carbon steel [35,36,37] and Cu-10Al-5Ni alloy [38], in hydrochloric acid solution [35], phosphoric acid [36], sulfamic acid [37] and sodium chloride [38] using electrochemical measurements. It was proven that NAC is an effective inhibitor of carbon steel corrosion, reaching an inhibition efficiency around 90%, in hydrochloric acid [35] and 87.6% for Cu-10Al-5Ni alloy corrosion in sodium chloride [38], lower than that of cysteine (96.4%) but higher than that of methionine (76.7%) [38]. For the carbon steel corrosion inhibition in phosphoric acid, both cysteine and acetylcysteine led to higher inhibition efficiency values than methionine and cystine [36]. In sulfamic acid, the trend remained almost unchanged and, therefore, inhibition efficiency of some amino acids containing sulfur atoms decreased in the order: *N*-acetylcysteine (97.3%) > cysteine (94.3) > S-benzylcysteine (92.7) > cystine (91.7) ≅ methionine (86.5) [37]. Also, K.F. Khaled et al. studied the 5LX60 steel corrosion inhibition in HCl solution using NAC, obtaining an efficiency value of 76.65% from the polarization resistance method, 71.86% from EIS and 67.94% from potentiodynamic polarization [39]. The average inhibition efficiency value obtained from the experiments was in good agreement with that of 74.6% obtained from theoretical calculations, applying an interdisciplinary, integrated quantitative model for predicting corrosion inhibition efficiency of amino acids [39].

In our previous studies, to investigate metal–inhibitor or drug–environment interactions, electrochemical measurements [40,41,42,43] and the DFT method were used [43,44].

In the current study, *N*-acetylcysteine (NAC) was investigated as an inhibitor for carbon steel corrosion in 1.0 mol L^−1^ hydrochloric acid solution, using potentiodynamic polarization (PDP) and electrochemical impedance spectroscopy (EIS), in a static regime, at room temperature. The experimental data obtained from both PDP and EIS were fitted according to the Temkin adsorption isotherm. The DFT method was selected as the quantum mechanical method for modeling the acetylcysteine molecule. The calculation of the global reactivity descriptors was carried out by the conceptual density functional theory methodology (CDFT). The theoretical investigation of the adsorption mechanism of acetylcysteine molecules on the Fe(110) crystal surface, in the presence of aqueous hydrochloric acid solution, was performed through an atomistic simulation based on a Monte Carlo approach with a statistical component. The computational study included *N*-acetylcysteine (NAC) in the category of the theoretically investigated amino acids that were reported by other authors, providing new data regarding its adsorption ability in hydrochloric acid solution.

## 2. Results and Discussion

### 2.1. Potentiodynamic Polarization

**Figure 1:**
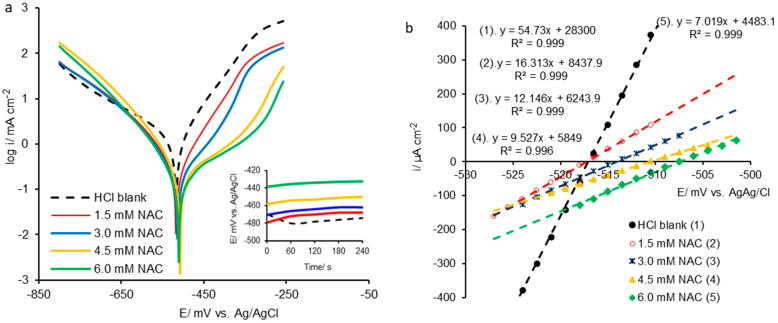
The potentiodynamic polarization results recorded for carbon steel corroded in hydrochloric acid solution, in the absence and presence of NAC inhibitor: (**a**)—semi-logarithmic polarization curves also displaying the measurements at the OCP; (**b**)—linear diagram obtained close to corrosion potential, in an overvoltage exploration domain from −10 mV to 10 mV.

The potentiodynamic polarization results are displayed in Figure 1, as Tafel polarization semi-logarithmic curves (Figure 1a) and a linear diagram processed in the potential range close to the corrosion potential (Figure 1b).

The addition of inhibitor in HCl solution leads to OCP displacement (inset graph in Figure 1a) in the positive direction, proportionally to NAC concentration, probably due to spontaneous inhibitor adsorption on the carbon steel surface, inducing the change in metal/electrolyte interface architecture and system balance improvement. The corrosion potential (E_corr_) insignificantly shifts in a positive direction, but the movement of the polarization curves in lower-current areas compared to that obtained in HCl blank solution is well highlighted (Figure 1a).

On the other hand, in the presence of NAC, the anodic polarization curves shift considerably towards higher potentials as the inhibitor concentration increases, having a significant impact on the anodic process, suggesting that the iron oxidation is delayed [1,13]. The cathodic polarization curves are almost overlapped up to the potential of −600 mV and then are located at higher potentials with respect to the blank solution polarization curve. Also, after −600 mV, Tafel straight lines on extensive potential segments are shown, indicating that the hydrogen evolution reaction is slowed down, especially at NAC concentrations higher than 1.5 mmol L^−1^ and, therefore, the inhibitor induces a limited effect on the cathodic process.

The semi-logarithmic polarization curves reveal that NAC acts as a mixed inhibitor, but predominantly anodic, inhibiting in a considerable manner the metal dissolution compared to the hydrogen evolution reaction.

The displacement of the anodic curves in the positive direction with the increase in the inhibitor concentration can also be associated with the development of an organic film on the carbon steel surface through the adsorption of NAC molecules possessing heteroatoms of oxygen, nitrogen and sulfur that facilitates their binding on the metal surface [1,11,45]. The unshared electrons of heteroatoms interact with the iron vacant d-orbitals from a metal network forming coordinative chemical bonds through which NAC molecules are adsorbed to the substrate [35,46]. The physical adsorption of NAC molecules via van der Waals forces could be another possibility for inhibitor binding on the carbon steel surface, or a mixed action mechanism between the chemical and physical adsorption can occur [1,35].

Moreover, the shifting of the polarization curves recorded in the presence of NAC towards lower-current areas in relation to the blank solution indicates that the corrosion current density (i_corr_) follows the same trend, decreasing with increasing inhibitor concentration. 

Also, NAC induces effects on the carbon steel polarization resistance (R_p_) highlighted by the diagram in Figure 1b, showing the current density linear dependence on potential over limited areas of overvoltages between ±10 mV, practically around the corrosion potential (E_corr_).

The electrochemical parameters, corrosion rate (CR) and inhibition efficiency (IE) are presented in Table 1. The average inhibition efficiency (IE_m_) was calculated as the arithmetic mean of the inhibition efficiencies obtained according to Equations (4)–(6).

Analyzing the potentiodynamic polarization data displayed in Table 1, the following conclusions are noted: (i) i_corr_ and CR decreased while R_p_ and IE increased with increasing NAC concentration; (ii) inhibition efficiency reached the highest level (IE_m_ = 87.8%) at an inhibitor concentration of 6.0 mmol L^−1^; (iii) the anodic Tafel slopes (b_a_) increased, suggesting that the inhibition process takes place by blocking the active sites on the carbon steel surface through NAC adsorption; (iv) thus, a protective layer was formed that interposed at the electrode/electrolyte interface, leading to corrosion process regression; (v) the cathodic Tafel slopes (b_c_) show almost similar values starting with the concentration of 3.0 mmol L^−1^, indicating that, in the presence of NAC, the hydrogen evolution reaction is attenuated, being less influenced by its concentration.

Ekemini B. Ituen et al. [35] studied the inhibition efficiency of NAC on the corrosion of X80 steel in 15% HCl solution using potentiodynamic measurements and electrochemical impedance spectroscopy (EIS), identifying a similar behavior of NAC. Thus, the potentiodynamic polarization revealed that [35]: (a) NAC acts as a predominantly anodic mixed inhibitor; (b) NAC addition leads to a decrease in the corrosion current density as its concentration increases; (c) NAC acts by adsorption on the steel surface, forming a protective film delaying the corrosion processes; (d) the inhibition efficiency increases with the inhibitor concentration, reaching the value of 91.1%, at 30 °C, at NAC concentration of 1.0 mmol L^−1^.

Also, Abd El-Hafez and Badawy [38] studied the corrosion inhibition of Cu–10Al–5Ni alloy in 3.5% NaCl solution using *N*-acetylcysteine that reached an inhibition efficiency of 87.6%, at the concentration of 6 mmol L^−1^.

### 2.2. Electrochemical Impedance Spectroscopy (EIS)

**Figure 2:**
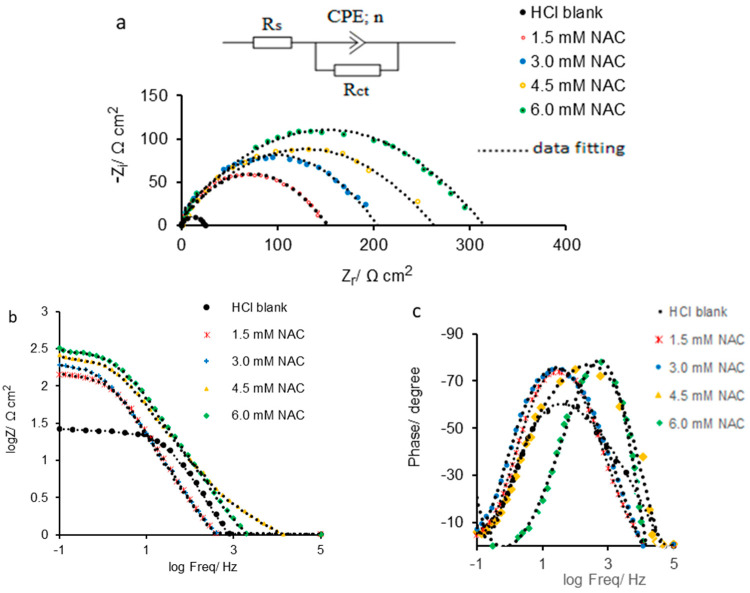
The results of EIS measurements on carbon steel immersed in 1.0 mol L^−1^ HCl solution, in the presence and absence of various NAC concentrations: (**a**)—the recorded Nyquist plots and equivalent circuit model; (**b**)—Bode impedance; (**c**)—Bode phase diagram.

The impedance response of the studied systems in the frequency range from 10^5^ Hz to 10^−1^ Hz was processed as Nyquist and Bode plots shown in Figure 2. The Nyquist diagram acquired for carbon steel immersed in HCl solution without and with various NAC concentrations is presented in Figure 2a, which also displays the employed equivalent circuit. As shown in Figure 2a, the capacitive loops deviating from a semi-circular shape were obtained for carbon steel in HCl solution without and with NAC inducing corrosion inhibition.

The deviation of the capacitive loops could be attributed to certain surface defects and/or heterogeneities at its level due to the random agglomeration of the chemical species at the metal/electrolyte interface [16]. Also, more extensive capacitive loops were recorded as the concentration of NAC increases, leading to the charge transfer resistance (R_ct_) gradually rising [1], indicating that the protective ability of the inhibitor against corrosion is more intense as its concentration reaches a higher value.

The Nyquist diagram (Figure 2a) was evaluated by fitting the experimental data using the Randles equivalent circuit consisting of charge transfer resistance (R_ct_) connected in parallel position with the constant phase element (CPE), both being linked in series with the solution resistance (R_s_).

The intersection of the capacitive loop with the real axis, at very low frequencies, represents (R_ct_ + R_s_), and at very high frequencies corresponds to the electrolyte resistance (R_s_) [47]. 

Generally, the constant phase element (CPE) replaces C_dl_ to highlight the protective layer non-ideal capacitance and to account for the surface inhomogeneity [1].

The CPE impedance (Z_CPE_) can be calculated using Equation (1) [22,38,41,48,49]:

(1) $$Z_{\mathrm{CPE}}=\frac{1}{T{\left(j\omega \right)}^{n}}$$

where: T is a proportional factor; j is the mathematical imaginary number (j^2^ = −1); ω is the angular frequency; n is a measure of surface irregularity; 0 ≤ n ≤ 1; for n = 0, Z_CPE_ represents a resistance, R = T^−1^; when n = 1, Z_CPE_ is a capacitance, C = T.

Consequently, when n is very close to unity, the CPE obeys the capacitive behavior [38], as shown in Table 2, where electrochemical parameters obtained from EIS are listed. 

The charge transfer resistance (R_ct_) was used to calculate the NAC inhibition efficiency, from EIS, according to Equation (2) [18].

(2) $$\mathrm{IE}=\frac{R_{\mathrm{ct}}-R_{\mathrm{ct}}^{o}}{R_{\mathrm{ct}}}\times 100$$

where: $R_{\mathrm{ct}}^{o}$ represents the charge transfer resistance obtained after carbon steel corrosion in 1.0 mol L^−1^ HCl blank solution; R_ct_ is the charge transfer resistance of carbon steel corroded in 1.0 mol L^−1^ HCl containing various NAC concentrations. 

Analyzing the Bode impedance diagram (Figure 2b), it can be seen that the impedance response at the frequency of 10^−1^ Hz (log Freq = −1) follows the same trend as in the Nyquist diagram. Thus, for the HCl blank solution, logZ has the lowest value (1.43), gradually increasing to 2.5 for the NAC concentration of 6 mmol L^−1^. As shown Table 2, for the impedance (Z), values almost similar to those from the Nyquist plot were identified which confirms the validity of the equivalent circuit used to fit the experimental data.

Classical Bode phase diagrams (Figure 2c) were obtained, consistent with the Nyquist ones displaying a single loop, highlighting for carbon steel immersed in HCl blank solution a phase angle maximum centered around −61.85 degrees in a low-frequency area. In the presence of the inhibitor, the phase angle maximum, located at the same frequency, shifts in the negative direction, reaching almost similar values between −75 and −74 degrees, for the NAC concentration of 1.5 mmol L^−1^ and 3.0 mol L^−1^, respectively, suggesting that, at the metal/environment interface, an upper layer was modeled by the adsorption of NAC molecules, which changed the carbon steel surface architecture. 

At inhibitor concentrations of 4.5 mmol L^−1^ NAC and 6.0 mmol L^−1^, respectively, the phase angle maximum is positioned at a higher frequency than those previously specified, meaning that the configuration of the adsorbed protective coating was slightly changed. Probably, a denser and coherently organized surface layer developed compared to the one adsorbed at lower concentrations, strongly restricting the ion exchange at the electrode/electrolyte interface [50].

As can be seen from Table 2, R_ct_ increases considerably, and the NAC inhibition efficiency is slightly higher than previously obtained, probably due to the development of a more compact upper layer, taking into account that the inhibitor action takes place on a surface with the morphology completely changed during potentiodynamic polarization. The layer organized on the surface improves the corrosion protection, ensuring a substantial blocking of the surface-active sites, leading to the NAC inhibition efficiency increasing.

Also, the decline in R_s_ and C_dl_ is observed, due to the capacity of NAC molecules to displace preadsorbed molecules of water and/or other ions [1], such as chloride anions. Chi-squared test (χ^2^ test) values are lower than 0.001 and increase with increasing inhibitor concentration.

The results obtained from both electrochemical methods are in good agreement, with the inhibition efficiency from EIS reaching a value of 90.7% compared to that calculated from potentiodynamic polarization of 87.8%, at 6.0 mmol L^−1^ NAC concentration, in HCl solution.

Some additional clarifications are needed regarding the differences between the inhibition efficiencies obtained from PDP and EIS. First of all, it is observed that with the increase in NAC concentration, the difference decreases, reaching the value of 2.9%, at 6 mmol L^−1^ NAC, which is included within the allowed limit. Also, it is known that alternative current reduces the risks and phenomena associated with electrochemical corrosion. Generally, under the same condition, higher polarization resistance is determined from EIS compared to the one obtained from PDP (especially in the presence of inhibitor) and, implicitly, the inhibition efficiency increases.

More or less hypothetically, the orderly molecular arrangement of NAC molecules on the substrate during EIS leads to the formation of an even layer, and the inhibitor desorption risk is reduced. During PDP, a random distribution of NAC molecules on the surface can take place and the coating can be disturbed by the occurrence of certain microcracks, that stimulate corrosion, especially at low inhibitor concentrations. 

Ekemini B. Ituen et al. [35] used the same equivalent circuit to fit the data corresponding to X80 steel corroded in 15% HCl solution containing various NAC concentrations, obtaining from EIS a NAC inhibition efficiency of 93.1%, at 1.0 mmol L^−1^ inhibitor concentration.

Also, Ghada M. Abd El-Hafez et al. [38] showed that the Cu/NaCl electrolyte interface with an adsorbed inhibitory film behaves like an ideal capacitor, obtaining for n values close to unity, meaning that it was not necessary to replace C_dl_ with CPE. The results obtained were valid for three amino acids, namely, cysteine, methionine and *N*-acetylcysteine (NAC), studied as corrosion inhibitors for copper in 3.5% NaCl solution.

### 2.3. Adsorption Isotherm Approach

To quantitatively express the adsorption of the NAC molecules on the carbon steel surface, an appropriate model of an adsorption isotherm must be applied, by fitting the degree of surface coverage (θ) values, with maximum regression coefficients (R^2^). 

The degree of surface coverage (θ) was determined as IE/100 [16,38,42,51], taking into consideration its average values obtained from the potentiodynamic polarization (PDP) and, also, from the electrochemical impedance spectroscopy (EIS). 

**Figure 3:**
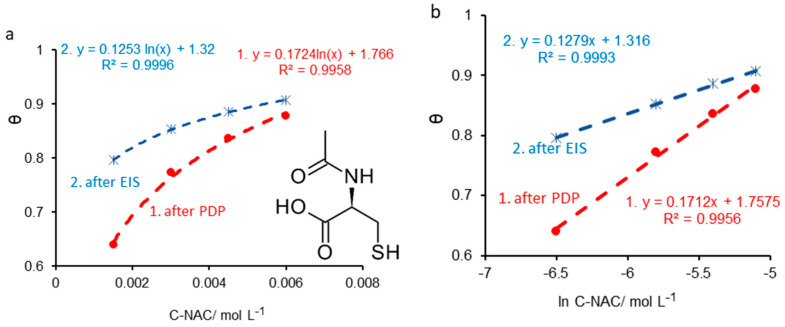
Simulation of surface coverage degree calculated from PDP and EIS in relation to *N*-acetylcysteine (NAC) concentration (**a**); Temkin isotherm applied for carbon steel immersed in 1.0 mol L^−1^ HCl solution containing various NAC concentrations, after both PDP and EIS (**b**).

Primarily, the evolution of the surface coverage degree (θ) was followed depending on the concentration of NAC. As the equations in Figure 3a show, after both the potentiodynamic polarization (PDP) and the electrochemical impedance spectroscopy (EIS), the surface coverage degree displays a logarithmic tendency in relation to NAC concentration. The confidence coefficients (R^2^) reached values very close to unity, namely, 0.9958 (PDP) and 0.9996 (EIS), meaning that θ = f [ln(C − NAC)] represents a straight line, just as the linearized form of the Temkin isotherm requires (Equation (6)). Note that, in this study, the Temkin isotherm is valid for inhibitor concentrations lower than 10^−2^ mol L^−1^.

According to the Temkin adsorption isotherm, θ is dependent on the inhibitor concentration as shown in Equation (3) [51].

(3) $$e^{-2\alpha \theta }=K\cdot C$$

Equation (3) is logarithmized and, after rearranging the terms, the linearized form of the Temkin adsorption isotherm is obtained (Equation (4)).

(4) $$\theta =-\frac{1}{2\alpha }\mathrm{lnC}-\frac{1}{2\alpha }\mathrm{lnK}$$

where: K (L mol^−1^) represents the adsorption–desorption constant; α is a factor describing the inhibitor interactions with the substrate; the positive value of α indicates an attractive behavior of the inhibitor for the substrate; if α is negative, it suggests substrate repulsion for layer adsorption [51].

The parameter f is defined as follows (Equation (5)) [51]:

f = −2α(5)

Accordingly, the Temkin adsorption isotherm can be written as Expression (6) [42,51]:

(6) $$\theta =\frac{1}{f}\mathrm{lnC}+\frac{1}{f}\mathrm{lnK}$$

The sign of the parameter f is opposite to that of α. If α < 0, f > 0, it involves the repulsion of inhibitor molecules; if α > 0, f < 0, the attractions take place [51]. Also, f represents a positive factor that characterizes the surface heterogeneity [52].

Equation (6) represents another form of the Temkin adsorption isotherm and, as specified in the previous paragraphs, by plotting θ = f [ln (C − NAC)] a straight line is obtained, with a slope equal to 1/f and the intercept [(1/f)lnK], from which K is easily deduced [51]. The values of the linear correlation coefficients are very close to unity, as shown the equations inserted in Figure 3b.

Consequently, K was determined with Equations (7) (for PDP) and (8) (for EIS).

(7) $$K_{\mathrm{PP}}=e^{1.7575\cdot f}\;$$

(8) $$K_{\mathrm{EIS}}=e^{1.316\cdot f}\;$$

The standard free adsorption energy (${\Delta G}_{\mathrm{ads}}^{o}$) was determined using Equation (9) [16,38,42,51].

(9) $${\Delta G}_{\mathrm{ads}}^{o}=-\mathrm{RTln}\left(55.5\cdot K\right)$$

where R is the universal constant of gases (8.31 J mol^−1^ K^−1^), T is the temperature (298 K) and 55.5 is the value of the molar concentration of water in the solution.

The ${\Delta G}_{\mathrm{ads}}^{o}$ value and other parameters deduced from the Temkin adsorption model are presented in Table 3.

From Table 3, it can be seen that from both methods, an almost similar value for ${\Delta G}_{\mathrm{ads}}^{o}$ is obtained, indicating that the NAC molecules adsorbed on the carbon steel surface form an adherent and stable adsorbed layer that regresses the corrosion processes.

Considering that the required threshold for chemical adsorption is −40 kJ mol^−^^1^, for ${\Delta G}_{\mathrm{ads}}^{o}$ [38,42,51], the negative value obtained, around −35 kJ mol^−^^1^, indicates a spontaneous adsorption via a NAC mixed action mechanism, predominantly chemisorption accompanied by physisorption that can take place through van der Waals forces, hydrogen bonds and/or Cl^−^ bridges. 

The high value of K implies that NAC is an efficient inhibitor for carbon steel corrosion in HCl solution due to the occurrence of strong interactions between ions from the electrical double layer and the inhibitor molecules [51]. 

The negative value of α can reveal that, on the metal surface, there are certain less reactive areas from the entropic point of view [52] and the average attraction forces of inhibitor molecules are weaker than those corresponding to the much more reactive surface places. The positive value of f confirms the surface heterogeneity and practically the existence of its entropically disadvantaged fractions that can further constitute anodic zones, where the corrosion processes take place with relatively high intensity.

Other adsorption isotherms were also analyzed, and the results obtained are shown in Table 4. It can be observed that the fitting of the experimental data was achieved with lower linearity coefficients (Freundlich, Flory–Huggins and El-Awady’s adsorption model, Frumkin) compared to those provided by the Temkin isotherm and, moreover, inconclusive values were obtained for K (Freundlich). Additionally, for PDP, the Frumkin isotherm can be applied if the last concentration is excluded. The experimental data accurately obeyed the first linearized form of the Langmuir isotherm (R^2^ close to unity) but with slopes of 0.98 and 1.048, respectively. As Kokalj showed in a 2023 study [52], if the slope deviates from unity the applicability of the Langmuir adsorption isotherm is limited. Furthermore, distinct values for K are obtained from the two Langmuir linearized equations, which also induce reluctance to apply this model for NAC adsorption on the carbon steel surface in hydrochloric acid solution (under the conditions stipulated in this study).

### 2.4. Quantum Chemical Analysis

The structural and electronic properties of the acetylcysteine (NAC) molecule determine its iron corrosion inhibition activity, these being responsible for the nature and intensity of the spontaneous interaction of the inhibitor molecule with the atoms on the metal surface. The density functional theory (DFT) model based on the Hohenberg–Kohn theorems allows a computational calculation whose results accurately describe the structure, energy and molecular properties of the theoretically investigated acetylcysteine.

**Figure 4:**
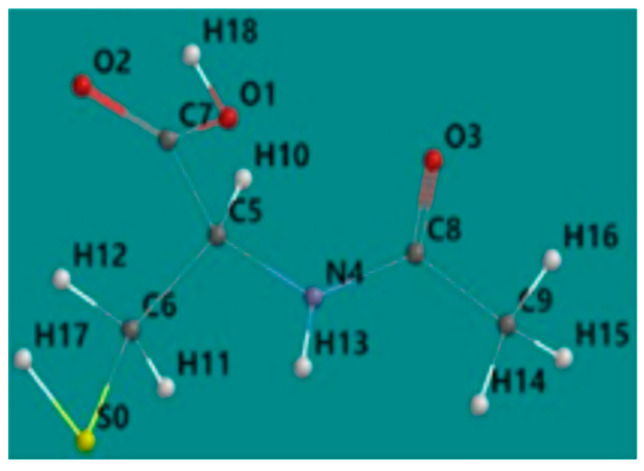
Equilibrium geometry in the aqueous phase of the acetylcysteine molecule in the singlet fundamental state.

As stated in the Methods section, the equilibrium geometry of the acetylcysteine molecule in the singlet fundamental state, shown in Figure 4, was determined by the DFT method.

The energy corresponding to the optimized geometry of the NAC molecule was validated as an authentic minimum located on the potential energy hypersurface by calculating the frequencies corresponding to the vibration modes. No values belonging to the set of complex numbers were obtained: the Hessian index value of zero was calculated, the values of all force constants being positive. The simulation of the vertical ionization energy in the first stage of the acetylcysteine molecule and the vertical electron affinity was carried out under the assumption of the validity of the Koopmans theorem by using the Kohn–Sham formalism.

The vertical ionization energy (I) and the vertical electron affinity (A) were approached in terms of the energies of the frontier molecular orbitals, known as highest occupied molecular orbital (HOMO) and lowest unoccupied molecular orbital (LUMO), as shown in Equations (10) and (11) [53].

The calculation was based on the eigenvalues of the virtual Kohn–Sham orthogonalized orbitals.

I = −E_HOMO_ (10)

A = −E_LUMO_ (11)

The DFT study indirectly provides information on the nature of the interactions between the acetylcysteine molecules and the metal surface atoms, since donor–acceptor interactions, if these are possible, involve the transfer of electrons between the NAC molecule frontier molecular orbitals and the d-vacant or p-occupied orbitals of the iron.

The distribution of the HOMO indicates the molecule sites for which the probability to donate electrons is high, while the ones that have a high chance to receive electrons are highlighted by the distribution of the LUMO. 

Therefore, the theoretical investigation supposed the shape calculation of the frontier molecular orbitals and the energy parameters such as the energy of the highest occupied molecular orbital (E_HOMO_), the energy of the lowest vacant molecular orbital (E_LUMO_), the energy gap between LUMO and HOMO (ΔE) calculated with Equation (12) [4,22,53], the vertical ionization energy in the first stage (I) and the vertical electron affinity (A).

**Figure 5:**
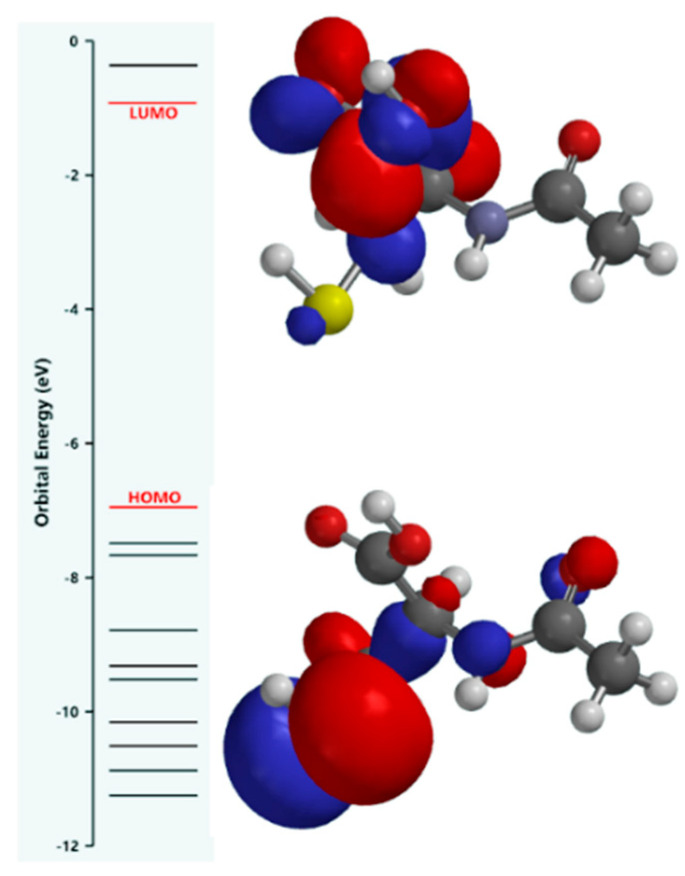
Shape of the HOMO and LUMO frontier molecular orbitals of acetylcysteine.

The obtained results presented in Figure 5 and Table 5 suggest that the acetylcysteine molecule has a predominantly nucleophilic character.

ΔE = E_LUMO_ − E_HOMO_ (12)

Generally, in the literature [4,22,53] it is stated that a greater E_HOMO_ value reveals a high electron-donating tendency of the inhibitor molecules towards an acceptor, leading to the increase in the inhibition efficiency [4]. Moreover, a lower value of E_LUMO_ shows that the inhibitor molecules have the ability to accept electrons from a donor [4,22,53]. Also, ΔE refers to the stability and reactivity of the molecule [4], in the sense that a small energy gap denotes a high reactivity of the molecules towards the metal surface [4], leading to the improvement of the adsorption process [4].

Thus, the predominant nucleophilic nature of the NAC molecule is defined by the following features: (i) there is a high probability that the electrons of the HOMO are donated to the iron atom that behaves as an electrophile having vacant d-orbitals. It must also be taken into account that the high energy of the acetylcysteine HOMO is strongly distributed on the sulfur atom of the thiol group but poorly localized on the nitrogen atom of the amino group and on the oxygen of the acetyl group; (ii) the electrons accommodated in the HOMO are readily available to form coordinative bonds, possessing the highest energy and the weakest bonds; (iii) the low energy value of the LUMO suggests that the transfer of electrons from occupied p-orbitals of the iron to the acetylcysteine molecule is also likely, due to most of the LUMO being located on the carboxyl group and only a small part being distributed on the thiol group; (iv) a stronger localization of the HOMO on the sulfur atom of the thiol group compared to that of the LUMO on the carboxyl group; (v) the relatively small energy gap between the HOMO and LUMO correlates with a significant reactivity of the acetylcysteine molecule, explained by the electronic density slightly changing by electron transfer.

As Table 5 shows, through analogy with the data reported by other authors for different amino acids [4,22,27], NAC has a higher reactivity than phenylalanine, aspartic acid and serine and lower reactivity than cysteine, tryptophan and tyrosine. As the ΔE value deceases, the inhibitor’s ability to attach to the substrate increases and, practically, its molecules are more easily adsorbed on the metal surface [4]. Thus, the adsorption ability of the inhibitors shown in Table 5 increases, as follows:

Aspartic acid< serine < phenylalanine < NAC < cysteine < tyrosine < tryptophan

**Figure 6:**
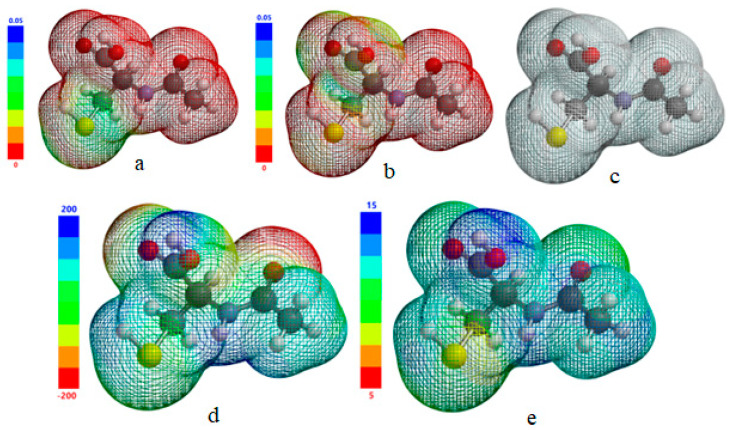
Electron density distribution of HOMO (**a**); electron density distribution of LUMO (**b**); total charge density isosurface simulated for the acetylcysteine molecule (**c**); simulated molecular electrostatic potential map (**d**); simulated local ionization potential map (**e**).

Even clearer arguments regarding the probability of the electron transfer direction in donor–acceptor type interactions were obtained by simulating the electronic density distribution of frontier molecular orbitals, the result being shown in Figure 6a,b. 

Also, frontier molecular orbitals were mapped onto the isosurface of total charge density (Figure 6c) calculated for the acetylcysteine molecule. Visual analysis of the isosurface maps of the frontier molecular orbitals confirms that the acetylcysteine molecule behaves globally as a nucleophile due to the transfer of electrons from its molecule to the iron atoms that probably takes place. 

More precise localization of the sites of the electron-rich molecule was achieved by simulating the 3D map of the electron density effective distribution in the acetylcysteine molecule (Figure 6d), mapping the molecular electrostatic potential onto the isosurface of the total charge density. As the relative color scale highlights, the nucleophilic centers are associated with regions of the molecule with high electron density, corresponding to negative values of the molecular electrostatic potential marked in red. Also, acetylcysteine molecule planarity is revealed that implies the parallel orientation of the inhibitor molecules with the metal surface, thus maximizing the intensity of attractive interactions and the area of the metal surface protected against corrosion.

The susceptible regions of the acetylcysteine molecule that easily donate electrons to the iron vacant d-orbitals are highlighted on the simulated map of the local ionization potential (Figure 6e), being associated with low values. In our previous study [43], based on the spatial distribution of the electrons and characteristic map of the molecular electrostatic potential, the performance of the drug metronidazole as a corrosion inhibitor for copper in hydrochloric acid solution was theoretically analyzed.

**Figure 7:**
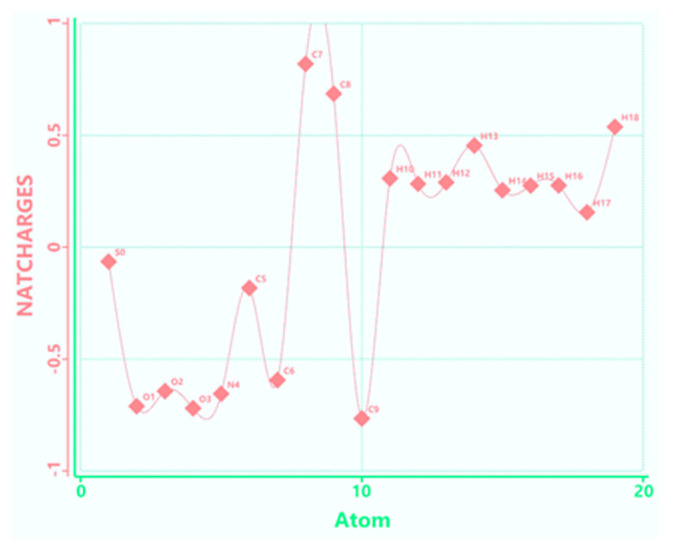
Plot of the natural atomic charge distribution in the acetylcysteine molecule.

The calculation of the partial atomic charges (Figure 7) by the natural bond orbital (NBO) method [27] allowed the identification of the active centers (atoms having significant negative partial charges) of the acetylcysteine molecule in the adsorption process on the iron surface, which were suggested by visual analysis of the difference in charge distribution on the 3D molecular surface. 

The nucleophilic centers of the acetylcysteine molecule are associated with oxygen atoms (O1, O2, O3), a nitrogen atom (N4) and carbon atoms (C6 and C9) and are identifiable in Figure 4. Additionally, around the sulfur atom, a partially negative charge is shown, contributing to the adsorption process of NAC molecules on the carbon steel surface. 

**Figure 8:**
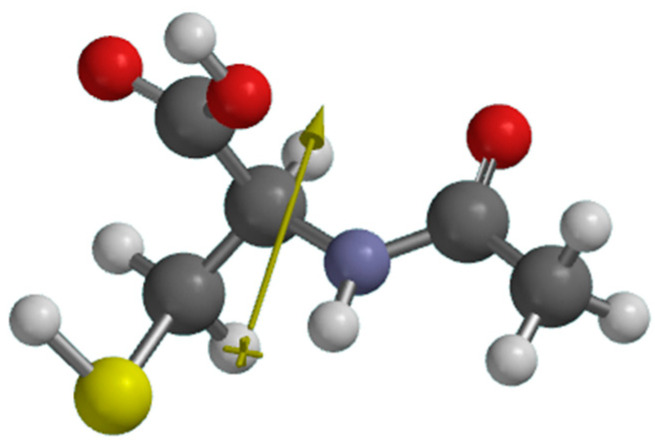
Simulated electric dipole orientation of acetylcysteine.

The simulation of the acetylcysteine molecule electric dipole moment (μ) allowed us to test the hypothesis relating to the occurrence of some electrostatic interactions at the metal/inhibitor interface. The obtained value of 3.98 D expresses an appreciable partial separation of the electric charge in the acetylcysteine molecule (Figure 8). Therefore, attractive interactions between acetylcysteine dipoles and chloride anions on the metal surface can take place. Also, electrostatic interactions between the Fe^2+^ ions in the electric double layer and the polar molecules of acetylcysteine are possible.

A relatively high value of dipole moment (~4 D) denotes a good adsorption of NAC on the carbon steel surface. On the other hand, a low ΔE value and high μ value show a good corrosion inhibition efficiency [53] of acetylcysteine. 

The calculation of global reactivity descriptors provides additional clues regarding the nature and intensity of interactions at the metal/inhibitor interface. According to the literature data, the main descriptors were determined for the NAC, as follows:

χ (electronegativity) indicating the atom’s power to attract electrons [4,11,22,27,43,44,53];
ε (chemical potential) representing the opposite of electronegativity (ε = −χ) [43,44];
η (hardness) is the descriptor regarding both molecule stability and its reactivity, being a measure of an atom’s resistance to charge transfer [4,11,22,27,43,44,53];
σ (softness) representing the hardness inverse and predicting the molecule reactivity (σ = 1/η) [4,22,43,44];
ω (electrophilicity index) indicating the molecule’s predisposition to accepting electrons [44].

Also, new descriptors were calculated (ω− and ω+) to predict the greater ability to donate and accept electrons, respectively.

ω− (electrodonating power);
ω+ (electroaccepting power);
ΔN (the fraction of the transferred electron), indicating the number of electrons transferred in the inhibitor/metal interaction [4,22,27,43,44,53].

The values of χ and η were calculated according to Koopmans’s theorem relating to the energy of frontier molecular orbitals (E_HOMO_ and E_LUMO_) using Equations (13) and (14), respectively [4,11,22,27,43,44,53].

(13) $$\chi =\frac{I+A}{2}\approx -\frac{E_{\mathrm{LUMO}}+E_{\mathrm{HOMO}}}{2}$$

(14) $$\eta =\frac{I-A}{2}\approx \frac{E_{\mathrm{LUMO}}-E_{\mathrm{HOMO}}}{2}$$

To determine the values of ω, ω_−_ and ω_+_, Equations (15)–(17) were used [44].

(15) $$\omega =\frac{\mu^{2}}{2\eta }=\frac{\chi^{2}}{2\eta }$$

(16) $$\omega_{-}=\frac{{\left(3I+A\right)}^{2}}{16\left(I-A\right)}=\frac{{\left(-3E_{\mathrm{HOMO}}-E_{\mathrm{LUMO}}\right)}^{2}}{16\left(E_{\mathrm{LUMO}}-E_{\mathrm{HOMO}}\right)}$$

(17) $$\omega_{+}=\frac{{\left(I+3A\right)}^{2}}{16\left(I-A\right)}=\frac{{\left(-E_{\mathrm{HOMO}}-3E_{\mathrm{LUMO}}\right)}^{2}}{16\left(E_{\mathrm{LUMO}}-E_{\mathrm{HOMO}}\right)}$$

ΔN values were calculated according to Equation (18) [4,22,27,43,44,53].

(18) $${\left(\Delta N\right)}_{\mathrm{NAC}-\mathrm{Fe}\left(1\;1\;0\right)}=\frac{\varphi_{\mathrm{Fe}}-\chi_{\mathrm{NAC}}}{2\left(\eta_{\mathrm{Fe}}+\eta_{\mathrm{NAC}}\right)}$$

where:${\;\varphi }_{\mathrm{Fe}}$ and $\chi_{\mathrm{NAC}}$ are the electronegativity of iron and the *N*-acetylcysteine (NAC) molecule, respectively; $\eta_{\mathrm{Fe}}$ and $\eta_{\mathrm{NAC}\;}$ are hardness of iron and the NAC molecule, respectively; $\varphi_{\mathrm{Fe}}$ represents the work function of an Fe(110) surface, being the best approximation of iron electronegativity, as it also quantifies the effect of electron–electron interactions.

For $\varphi_{\mathrm{Fe}\;}$ and $\eta_{\mathrm{Fe}\;}$ the values of 4.82 eV for Fe(1 1 0) and 0 eV mol^−1^ [27,54], respectively, were used in this study; the $\varphi_{\mathrm{Fe}}$ value resulted from the DFT calculation of A. Kokalj et al. [54]. 

The global reactivity descriptors are shown in Table 6.

Considering that higher electronegativity (χ) values denote a greater ability of molecules to accept electrons [4], NAC attracts electrons more strongly than cysteine. On the other hand, as the values of hardness (η) decrease and softness (σ) increase, the ability of inhibitor molecules to adhere to the metal surface increases and, consequently, they are more easily adsorbed [4].

Therefore, cysteine provides a higher adsorption ability and a higher global reactivity than acetylcysteine (NAC). However, the differences between the descriptor values are relatively small (especially softness), which means that both compounds can work as effective inhibitors for the carbon steel corrosion, in the presence of Cl− ions.

The fraction of the transferred electron (ΔN) is positive, indicating that the electrons are transferred from the inhibitor molecule to the metal surface [4] from acetylcysteine molecules to the vacant d-orbitals of iron.

The value of the electrophilicity index (ω) is in agreement with those of the electronegativity (χ), showing that the NAC molecules can accept electrons from the occupied p-orbital of iron, but the much too low value of electroaccepting power (ω_+_ = 0.962 eV) suggests a rather weak electrophile. 

The correlative analysis of the global reactivity descriptors shows that the acetylcysteine electronegativity is favorable to the energy change based on the appreciable deformability of the global electronic density, through an electron flow from acetylcysteine to the metal surface. Therefore, the behavior of the acetylcysteine molecule as a nucleophile is thermodynamically favored, explaining the positive values of the fraction of transferred electrons (ΔN) and the electrodonating power (ω_−_ = 4.887 eV) that is lower than the first step of vertical ionization energy, I = 6.95 eV (Table 5).

### 2.5. Monte Carlo Simulation

**Figure 9:**
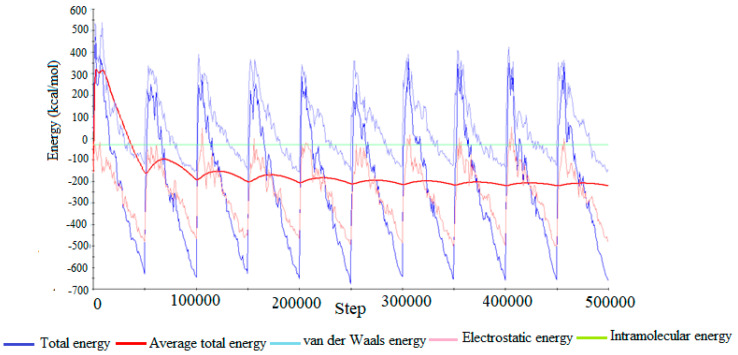
Profile of the total energy distribution during the search for the global minimum on the potential energy hypersurface.

The probabilistic Monte Carlo exploration of the configuration space of the simulated system, in order to identify the adsorption configuration characterized by the lowest energy, generated the total energy distribution shown in Figure 9.

From the analysis of Figure 9, it can be seen that the energy fluctuations corresponding to the long-range electrostatic interactions at the Fe(110)/acetylcysteine interface have much smaller amplitudes compared to the energy variations associated to the van der Waals interactions, denoting that physical adsorption in the atomistically simulated system is controlled by van der Waals forces.

**Figure 10:**
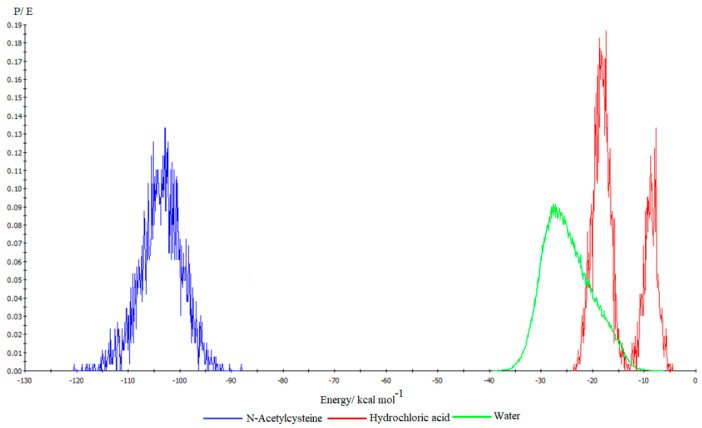
Adsorption energy distribution profile for the Fe(110)−HCl−55 H_2_O system.

The calculation of the adsorption energy through the simulated annealing procedure generated the adsorption energy distribution profile shown in Figure 10.

It can be stated that the adsorption of acetylcysteine molecules on the Fe(110) crystal surface is much stronger compared to the adsorption of water molecules and chloride ions. At equilibrium, the differential adsorption energy values calculated for acetylcysteine (−107.001 kcal mol^−1^), hydrochloric acid (−8.870 kcal mol^−1^) and water (−13.547 kcal mol^−1^) highlight the spontaneity of the adsorption process and confirm that acetylcysteine molecules strongly attach to the metal surface through covalent bonds, forming a stable protective layer.

Therefore, it can be assumed that the reduction of the interaction between the metal surface and the corrosive environment is the result of the progressive replacement of water molecules and ionic species preadsorbed on the metal surface by the acetylcysteine molecules.

**Figure 11:**
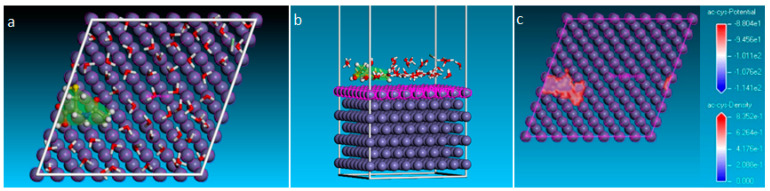
The adsorption configuration corresponding to the global minimum on the potential energy hypersurface: top view (**a**), side view (**b**); adsorption density field of NAC molecule on the Fe(110) substrate (**c**).

Figure 11a,b show the parallel orientation of the acetylcysteine molecule in relation to the metal surface at the end of the stochastic atomistic simulation, highlighting the active centers of the molecule involved in the interactions with the atoms on the metal surface.

From Figure 11b, it can be seen that the nucleophilic centers of the acetylcysteine molecule identified by the DFT calculation, which are associated with the atoms of O1, O2, O3, N4, C6 and C9, interact with the surface iron atoms and/or with Fe^2+^ ions from the electrical double layer formed at the electrode/electrolyte interface. The hypothesis of the existence of six adsorption centers is also confirmed by the volumetric representation of the force field density (Figure 11c), through which the covalent, long-range electrostatic and van der Waals interactions between the inhibitor and the metal surface can occur.

### 2.6. N-Acetylcysteine Adsorption Mechanism

The *N*-acetylcysteine (NAC) action mechanism is complex and several aspects highlighted by the experimental tests and theoretical calculations must be taken into account. From the Temkin adsorption isotherm, a high adsorption–desorption constant (K), around 29,000 L mol^−1^, was calculated and, therefore, the standard adsorption free energy (${\Delta G}_{\mathrm{ads}}^{o}$) of −35 kJ mol^−1^ was obtained. Consequently, NAC spontaneously adheres to the carbon steel surface through a mixed mechanism involving chemical adsorption accompanied by physical adsorption. Since ${\Delta G}_{\mathrm{ads}}^{o}$ reached a value relatively close to −40 kJ mol^−1^, it can be stated that the mechanism is a moderately chemical one, or chemical adsorption prevails over physical adsorption.

The DFT method revealed the NAC nucleophilic feature, and the calculation of the partial atomic charges by the NBO method pointed out six active centers concentrating substantial negative charges compared to other atoms.

Practically, the oxygen atoms, namely, O1, O2 and O3 from the carboxyl group (-COOH) and carbonyl group (>C=O), respectively, as well as the nitrogen atom (N4) of the amine group (>NH), provide the free electrons to the iron vacant d-orbital, thus forming chemical bonds between NAC molecules and the carbon steel surface, and in this way the pure-chemical adsorption takes place. The partial negative charge from around the sulfur atom also leads to the occurrence of NAC–substrate coordinative bonds by involving the unshared sulfur electrons and iron vacant d-orbital. Certain studies reported that sulfur was identified by SEM/EDS analysis on the carbon steel surface corroded in hydrochloric acid solution containing NAC [35].

The simulation of the acetylcysteine molecule electric dipole moment (μ) allowed us to test the hypothesis relating to the occurrence of some electrostatic interactions at the metal/inhibitor interface. The obtained value of 3.98 D expresses an appreciable partial separation of the electric charge in the acetylcysteine molecule (Figure 8). Therefore, attractive interactions between acetylcysteine dipoles and chloride anions on the metal surface can take place.

The charges concentrated at the carbon atom (C6) from the methyl group linked to the thiol (HS-CH_2_-) and C9 were located at the most negative level, which belongs to the methyl group linked to the carbonyl (acetyl group) and can lead to the appearance of long-range electrostatic interactions with Fe^2+^ ions from the electrical double layer, also shown by the Monte Carlo simulation (Figure 9).

According to Ahmed E. Fazary et al. [55], NAC can dissociate by releasing hydrogen from the carboxyl (-COOH) or thiolic (-SH) groups, forming a free ligand of the [NAC]^2−^ type, which can generate complexes with bivalent metal ions [55]. Therefore, the NAC complex with iron cations can occur, binding to the substrate through van der Waals bonds as highlighted by the Monte Carlo simulation.

Ekemini B. Ituen et al. [35] reported Fe-NAC complex formation, analyzing the spectral characteristics of acetylcysteine, displayed by UV–Vis spectrophotometry, before and after the immersing of a carbon steel sample in hydrochloric acid solution containing inhibitor. The change in the position and shape of the NAC spectrum, after the steel immersion, could be associated with the Fe-NAC complex, which adsorbs on the metal surface [35].

## 3. Materials and Methods

### 3.1. Materials

The carbon steel plates, with an active area of 1.0 cm^2^, were submitted to corrosion at room temperature (24 ± 1 °C) and a static regime, in 1.0 mol L^−1^ HCl blank solution and 1.0 mol L^−1^ HCl blank solution containing various concentrations of *N*-acetylcysteine (NAC): 1.5 mmol L^−1^; 3.0 mmol L^−1^; 4.5 mmol L^−1^; 6 mmol L^−1^. N-acetylcysteine, a Merck product (assay ≥ 99%), is a light-yellow powder and water-soluble. Low-carbon steel (OL 37 Romanian type) was used, containing: C < 0.22; Mn < 0.36%; Si < 0.35%; P < 0.06%; S < 0.06%%; the remainder being Fe up to 100%. Analytical purity hydrochloric acid from Merck and bi-distilled water were used to prepare the uninhibited and inhibited NAC solutions. Before corrosion, the samples were mechanically polished with emery paper, degreased with acetone and dried in warm air.

### 3.2. Methods

#### 3.2.1. Electrochemical Measurements

Both the potentiodynamic polarization and electrochemical impedance spectroscopy (EIS) were performed using a standard electrochemical cell with three electrodes coupled to a VoltaLab 40 potentiostat/galvanostat, with VoltaMaster 4 software, that allows the simultaneous evaluation of corrosion current density and corrosion rate (potentio-gravimetric method). The working electrode was constituted by a carbon steel plate with an area of 1.0 cm^2^, the auxiliary electrode was made from platinum plate (area of 1.0 cm^2^) and, as a reference, a Ag/AgCl electrode was used. The electrochemical assembly was reported in our previous studies [13,40,41,42,43,44]. 

#### 3.2.2. Potentiodynamic Polarization

The potentiodynamic polarization was carried out after 4 min of immersion time of the electrodes at OCP, with a potential scan rate of 1.0 mV s^−1^, in the potential range from −800 mV to −100 mV. The semi-logarithmic potentiodynamic curves were recorded in the potential range of ±250 mV with respect to the corrosion potential values. Also, the linear diagram was drawn close to the corrosion potential, in an overvoltage exploration domain from −10 mV to 10 mV.

By derivation of the equations (dy/dx) inserted in Figure 1b, where the y-axis represents the current density (i/μA) and x-axis the potential (E/mV), the slopes of the straight lines are obtained that are associated with the polarization resistance (R_p_), according to relations (19) and (20) [40,48].

(19) $$10^{-3}{\left(\frac{di}{dE}\right)}_{E\to E_{corr}}=\frac{1}{R_{p}}$$

(20) $$R_{p}={\left(\frac{di}{dE}\right)}_{E\to E_{corr}}^{-1}\times 10^{3}$$

The corrosion current density (i_corr_) was computed at the intersection of the anodic and cathodic Tafel lines extrapolated to the corrosion potential and, subsequently, its value was converted to corrosion rate (CR) using Equation (21) [13].

(21) $$\mathrm{CR}\;\left({\mathrm{mmY}}^{-1}\right)=\frac{i_{\mathrm{corr}}A}{1000\mathrm{zF}\rho }\times 24\times 365\times 10^{3}$$

where z is the number of electrons interchangeable in the process; i_corr_ is the corrosion current density (A m^−2^); A represents iron atomic mass (g mol^−1^); F is Faraday’s constant (A·h); ρ is iron density (kg m^−3^).

The electrochemical parameters, namely, the corrosion potential (E_corr_), corrosion current density (i_corr_), as well as its conversion to corrosion rate (CR), anodic and cathodic Tafel slopes (b_a_ and b_c_) and polarization resistance (R_p_), were computed using the VoltaMaster 4 software. The inhibition efficiency (IE/%) of NAC in the carbon steel corrosion, in 1.0 mol L^−1^ HCl solution, was determined taking into consideration the values of i_corr_ [35,38,40,47], CR [16] and R_p_ [16,40,47], before and after potentiodynamic polarization using Equations (22)–(24).

(22) $$\mathrm{IE}=\frac{i_{\mathrm{corr}}^{o}-i_{\mathrm{corr}}}{i_{\mathrm{corr}}^{o}}\times 100$$

(23) $$\mathrm{IE}=\frac{{\mathrm{CR}}^{o}-\mathrm{CR}}{{\mathrm{CR}}^{o}}\times 100$$

(24) $$\mathrm{IE}=\frac{R_{p}-R_{p}^{o}}{R_{p}}\times 100$$

where: $i_{\mathrm{corr}}^{o}$, CR^o^ and $R_{p}^{o}$ represent the corrosion current density, corrosion rate and polarization resistance recorded after carbon steel corrosion in 1.0 mol L^−1^ HCl blank solution; i_corr_, CR and R_p_ represent the corrosion current density, corrosion rate and polarization resistance computed for carbon steel corroded in 1.0 mol L^−1^ HCl solution containing various NAC concentrations. 

#### 3.2.3. Electrochemical Impedance Spectroscopy (EIS)

The electrochemical impedance spectroscopy (EIS) was carried out, after the potentiodynamic polarization, at the corrosion potential values, in the frequency range of 10^5^ Hz and 10^−1^ Hz, with an AC perturbation signal of 10 mV. The Nyquist and Bode diagrams were recorded for the carbon steel immersed in 1.0 mol L^−1^ HCl blank solution and 1.0 mol L^−1^ HCl solution containing various NAC concentrations.

### 3.3. Computational Details

The DFT method was selected as the quantum mechanical method for modeling the acetylcysteine molecule. The calculation of the global reactivity descriptors was performed using the conceptual density functional theory (CDFT) methodology. 

To understand the corrosion inhibition phenomenology, it is necessary to elucidate the mechanism by which the energetically favorable interactions between the acetylcysteine molecules and the metal surface are achieved. 

The SM8 model to simulate the continuous dielectric medium was used to calculate the equilibrium geometry of the acetylcysteine molecule in the singlet fundamental state, in the aqueous phase. The electronic structure and total energy were determined based on the Becke 3 Lee Yang Parr (B3LYP) hybrid model of the exchange-correlation function, using the Pople, double-zeta basis set, extended with diffusion and polarization functions, 6–31G+(d), for all atoms except the hydrogen ones.

The geometric optimization without symmetry restriction was performed in Cartesian coordinates at the restricted Hartree–Fock (RHF) level. The geometric optimization calculation was performed by the Pulay direct inversion in the iterative subspace (DIIS) procedure using the geometric direct minimization (GDM) optimization algorithm. Convergence acceleration was achieved by the Broyden–Fletcher–Goldfarb–Shanno (BFGS) approach in the iterative subspace. In this study, the reporting was carried out on the Fe(110) surface due to the fact that this could be a more stable plane than Fe(100) and Fe(111). 

Computational quantum mechanics calculations using the DFT method were performed with the Spartan 14 software application.

### 3.4. Monte Carlo Simulation

Unlike experimental investigations, the computational studies, based on the atomistic simulation techniques, provide information at the atomic and molecular level about the covalent and non-covalent interactions that take place at the metal/inhibitor interface. Thus, the Metropolis Monte Carlo atomistic simulation was chosen to reveal the adsorption configuration and interactions between acetylcysteine molecules and the metal surface using modeling by force fields of molecular mechanics. 

The theoretical investigation of the adsorption mechanism of acetylcysteine molecules on the Fe(110) crystal surface, in the presence of aqueous hydrochloric acid solution, was carried out through an atomistic simulation based on a Monte Carlo approach with a statistical component. Using the periodic frontier conditions to eliminate surface effects, the simulation of a representative part of the macroscopic phenomenon of inhibitor adsorption on the metal surface was performed in a computational cell with the following dimensions: 19.86 Å × 19.86 Å × 64.19 Å. The modeling of the Fe(110) supercell was carried out using 8 layers, each consisting of 84 Fe atoms.

The stochastic search, involving a Metropolis Monte Carlo algorithm, of the possible adsorption configurations was carried out in the configurational space corresponding to the system consisting of an acetylcysteine molecule, a hydrochloric acid molecule and 55 water molecules, being guided by a Boltzmann probability distribution that is dependent on both energy and temperature. 

The geometric optimization of all the components of the simulated system was performed using the Smart algorithm and the condensed phase optimized molecular potentials for atomistic simulation studies (COMPASS) force field with its assigned charges. The modeling of the electrostatic interactions was achieved by the Ewald and group summation method, and the atom-based summation method was selected for the calculation of the van der Waals interactions.

Monte Carlo simulation was carried out in the materials modeling and simulation environment Materials Studio 7.0.

## 4. Conclusions

The potentiodynamic polarization (PDP) showed that *N*-acetylcysteine (NAC) behaves as a predominantly anodic inhibitor for carbon steel corrosion, in 1.0 mol L^−1^ HCl solution acting by adsorption on the metal surface and that induces a high inhibition efficiency (IE) of 87.8%.

Electrochemical impedance spectroscopy (EIS) confirms the good adherence and stability of the adsorbed film on the carbon steel surface, providing a high protective ability against corrosion, around 90%, a value close to that obtained from potentiodynamic polarization.

The best fitting of experimental data obeyed the Temkin adsorption isotherm. The standard free energy of adsorption ($\Delta G_{ads}^{o}$) reached a value around −35 kJ mol^−1^, indicating that NAC acts through a mixed mechanism, with the chemical adsorption prevailing over the physical one. 

The computational study confirms the experimental results showing that the NAC’s spontaneous adsorption occurs mainly through covalent chemical bonds that prevail over physical adsorption controlled by van der Waals forces.

## Figures and Tables

**Table 1:** Electrochemical parameters, corrosion rate (CR) and inhibition efficiency (IE/%) recorded from potentiodynamic polarization accomplished for carbon steel, in 1.0 mol L^−1^ HCl solution, in the absence and in the presence of NAC, at room temperature.

C-NAC/ mol L^−1^	OCP/ mV	E_corr_/mV	i_corr_/ mA cm^−2^	b_a_/ mV dec^−1^	b_c_/ mVdec^−1^	CR/ mmY^−1^	R_p_/ Ω cm^2^	IE/%			IE_m_/%
								from Equation (4)	from Equation (5) cr	from Equation (6)	
0	−474 ± 4	−517.2 ± 2.5	1.13 ± 0.19	79.3	−159.3	13.3 ± 1.2	18.3 ± 1.2	-	-	-	-
1.5 × 10^−3^	−468 ± 3	−517.3 ± 2.1	0.44 ± 0.08	85.9	−110.2	5.2 ± 0.6	61.3 ± 2.2	61.1	60.9	70.1	64.0 ± 0.6
3.0 × 10^−3^	−462 ± 3	−514.1 ± 2.2	0.26 ± 0.08	113.3	−84.5	3.1 ± 0.4	82.3 ± 4.4	76.9	77.8	77.3	77.3 ± 0.4
4.5 × 10^−3^	−450 ± 2	−510.8 ± 1.9	0.19 ± 0.02	209.1	−85.6	2.2 ± 0.1	114.9 ± 4.2	83.2	83.5	84.1	83.6 ± 0.6
6.0 × 10^−3^	−432 ± 2	−507.5 ± 1.3	0.13 ± 0.02	213.2	−87.5	1.6 ± 0.1	142.5 ± 6.4	88.4	87.9	87.2	87.8 ± 0.3

**Table 2:** Electrochemical parameters and inhibition efficiency (IE) calculated from EIS for carbon steel corroded in 1.0 mol L^−1^ HCl solution, in the absence and presence of NAC.

C-NAC/ mol L^−1^	Nyquist Parameters					IE/%
	R_s_/ mΩ cm^2^	C_dl_/ μF cm^−2^	R_ct_/ Ω cm^2^	n	χ^2^ × 10^−4^	
0	403.3 ± 16.1	922.6 ± 36.9	28.6 ± 1.2	0.944	1.54	-
1.5 × 10^−3^	267.8 ± 10.7	816.8 ± 32.6	140.5 ± 5.6	0.987	1.86	79.64 ± 0.04
3.0 × 10^−3^	180.2 ± 8.5	650.1 ± 19.1	194.8 ± 9.3	0.978	2.48	85.3 ± 0.09
4.5 × 10^−3^	167.5 ± 9.2	316.3 ± 17.4	251.5 ± 13.8	0.969	3.36	88.6 ± 0.1
6.0 × 10^−3^	102.8 ± 5.1	257.4 ± 12.9	309.1 ± 15.5	0.97	6.22	90.7 ± 0.1
**C-NAC/** **mol L^−1^**	**Bode Parameters**					
	**log Z**			**Z/** **Ω** **cm^2^**	**Phase/degrees**	
0	1.43			26.9	−61.85	
1.5 × 10^−3^	2.15			142.8	−74.77	
3.0 × 10^−3^	2.29			194.9	−74.85	
4.5 × 10^−3^	2.41			257.0	73.97	
6.0 × 10^−3^	2.50			316.2	−75.65	

**Table 3:** Temkin parameters and ${\Delta G}_{\mathrm{ads}}^{o}$ value obtained from both PDP and EIS, for the carbon steel immersed in HCl solution containing various NAC concentrations.

Electrochemical Measurement	Slope	f	α	K/ L mol^−1^	R^2^	$\Delta G_{\mathrm{ads}}^{o}/$ kJ mol^−1^
PDP	0.1712	5.84	−2.92	28,566.8	0.9956	−35.3
EIS	0.1279	7.82	−3.91	29,436.8	0.9993	−35.4

**Table 4:** The results obtained by applying other adsorption isotherms.

Adsorption Isotherm	Linearized form	Slope		Intercept			
Freundlich	lnθ=nlnC+lnK lnθ = f(lnC)	n		lnK		R^2^	
		PDP	EIS	PDP	EIS	PDP	EIS
		0.2236	0.0947	1.0222	0.3904	0.9917	0.9868
Langmuir	$\frac{C}{\theta }=C+\frac{1}{K}$ C/θ = f (C)	1		1/K		R^2^	
		0.98	1.048	0.0009	0.0005	0.9954	0.996
	$\frac{\theta }{1-\theta }=\mathrm{KC}$ [θ/(1 − θ)] = f(C)	K		0			
		1180	1558	−0.5	0.77	0.9946	0.9735
Flory–Huggins	$\ln\frac{\theta }{C}=\mathrm{bln}\left(1-\theta \right)+\mathrm{lnK}$ ln(θ/C) = f(1 − θ)	b		lnK		R^2^	
		1.4076	1.5916	8.7072	7.8147	0.9506	0.9846
El-Awady’s model	$\ln\frac{\theta }{1-\theta }=\mathrm{ylnC}+\mathrm{lnK}$ $\ln\frac{\theta }{1-\theta }$ = f(lnC)	y		lnK		R^2^	
		0.9996	0.6670	7.0521	5.6759	0.9856	0.9915
Frumkin	$\ln\left[C^{-1}\left(\frac{\theta }{1-\theta }\right)\right]=2\alpha \theta +\mathrm{lnK}$ $\ln[C^{-1}\left(\frac{\theta }{1-\theta }\right)]=f\left(\theta \right)$	2α		lnK		R^2^	
		NA	−4.3513	-	11.322	-	0.9751

**Table 5:** Energy parameters of the *N*-acetylcysteine (NAC) molecule compared to those obtained for other reported amino acids.

Amino Acid	E_HOMO_/eV	E_LUMO_/eV	ΔE/eV	I/eV	A/eV	Reference
N-acetylcysteine	−6.95	−0.90	6.05	6.95	0.90	This study
Phenylalanine ^a^	−6.94	−0.431	6.48	-	-	[22]
Aspartic acid ^a^	−7.06	−0.314	6.741	-	-	[22]
L-cysteine ^b^	−6.745	−0.804	5.941	-	-	[4]
Serine ^c^	−7.15	−0.644	6.506	-	-	[27]
Tryptophan ^c^	−5.82	−0.811	5.009	-	-	[27]
Tyrosine ^c^	−6.36	−0.798	5.562	-	-	[27]

^a,b^ In the presence of Cl^−^/aqueous solution; ^c^ in water.

**Table 6:** Global reactivity descriptors calculated for *N*-acetylcysteine (NAC) compared to those of cysteine.

Nr.crt.	Descriptor	N-Acetylcysteine	Cysteine
1	χ/eV	3.925	3.772 [4]
2	ε/eV	−3.925	-
3	η/eV	3.025	2.971 [4]
4	σ/(eV)^−1^	0.331	0.337 [4]
5	ΔN	0.148	0.543 [4]
6	ω/eV	2.546	-
7	ω_−_/eV	4.887	-
8	ω_+_/eV	0.962	-

## Data Availability

Not applicable.

## Sample Availability

Samples of the compounds are not available from the authors.

## Abbreviations

NAC	N-acetylcysteine
PDP	potentiodynamic polarization
EIS	electrochemical impedance spectroscopy
OCP	open circuit potential
i_corr_	corrosion current density
E_corr_	corrosion potential
R_p_	polarization resistance
CR	corrosion rate
b_a_, b_c_	anodic and cathodic Tafel slopes
R_ct_	charge transfer resistance
C_dl_	double-layer capacitance
R_s_	solution resistance
IE%	inhibition efficiency
K	adsorption–desorption constant
$\Delta G_{ads}^{o}$	standard free adsorption energy
θ	degree of surface coverage
χ	electronegativity
ε	chemical potential
η	hardness
σ	softness
ω	electrophilicity index
ω−	electrodonating power
ω+	electroaccepting power
ΔN	fraction of the transferred electron
